# The Effects of Mesenchymal Stem Cells on Oral Cancer and Possible Therapy Regime

**DOI:** 10.3389/fgene.2022.949770

**Published:** 2022-06-30

**Authors:** Tong Yang, Shuai Tang, Shan Peng, Gang Ding

**Affiliations:** School of Stomatology, Weifang Medical University, Weifang, China

**Keywords:** mesenchymal stem cells, oral cancer, tumor microenvironment, therapy, delivery vehicles

## Abstract

Mesenchymal stem cells (MSCs) are characterized by self-renewal, rapid proliferation, multipotent differentiation, and low immunogenicity. In addition, the tropism of MSCs towards injured tissues and tumor lesions makes them attractive candidates as cell carriers for therapeutic agent delivery and genetic material transfer. The interaction between tumor cells and MSCs in the tumor microenvironment plays an important role in tumor progression. Oral cancer is one of the most common malignant diseases in the head and neck. Although considerable improvements in the treatment of oral cancer were achieved, more effective and safer novel agents and treatments are still needed, and deeper studies on the etiology, pathology, and treatment of the oral cancer are desirable. In the past decades, many studies have reported the beneficial effects of MSCs-based therapies in the treatment of various diseases, including oral cancers. Meanwhile, other studies demonstrated that MSCs may enhance the growth and metastasis of oral cancer. In this paper, we reviewed the research progress of the effects of MSCs on oral cancers, the underlying mechanisms, and their potential applications in the treatment of oral cancers.

## 1 Introduction

MSCs are a class of non-hematopoietic stem cells belonging to the mesoderm, with the characteristics of self-renewal, high proliferation, multi-directional differentiation potential. It has been demonstrated that one of the possible sources of MSCs is the blood vessel wall, which is a type of perivascular cells. This provides a great potential for its involvement in tissue regeneration (da Silva Meirelles et al., 2008). Many studies have been conducted to validate the use of MSCs in bone regeneration and nerve regeneration (Liau et al., 2020; Fu et al., 2021). MSCs exhibit chemotactic properties similar to immune cells in response to tissue injury and inflammation. MSCs can release various bioactive factors, such as immunosuppressive molecules, growth factors, chemokines, and complement components to regulate the inflammatory process and create a balanced inflammatory and regenerative microenvironment in damaged tissues, thereby treating various degenerative and inflammatory diseases (Shi et al., 2018). Because of their inherent ability to migrate and colonize into tumor tissues, MSCs were reported to closely interacted with tumor and tumor cells. It was shown that interleukin (IL)-12-expressing MSCs could inhibit the growth of tumor in a model of mouse renal cell carcinoma (Gao et al., 2010), and in a mouse melanoma brain metastasis model, intracarotid administration of oncolytic herpes simplex virus-armed MSCs significantly prolonged the survival of mice (Du et al., 2017). Thus, MSCs are attracting increasing interest in the field of oncology.

Oral cancer is one of the most common malignant tumors of the head and neck, often occurring in the middle-aged and elderly population and more than 90% of oral cancer are oral squamous cell carcinoma (OSCC) (Choi and Myers, 2008). Over the past decades, the incidence of oral cancer has been on a gradual increase. Smoking, excessive alcohol consumption as well as betel nut chewing, are the main causative factors of oral cancer. In addition, it cannot be ignored that the number of young patients suffering oral cancer also has been increasing. According to public reports, the number of new oral cancer cases and patients of new deaths reached 377,000 and 177,000, respectively, worldwide in 2020 (Chai et al., 2020; Grigolato et al., 2021; Sung et al., 2021).

A growing number of studies have demonstrated the beneficial effects of MSCs-based therapies in the treatment of various diseases, including oral cancers. However, some other studies showed that MSCs may enhance the growth and metastasis of oral cancer. The exact role of MSCs’ effects on tumor, i.e., whether MSCs could exert tumor-suppressive effects or, conversely, whether they favor tumor growth, has not been fully elucidated, and some concerns, including the mechanisms responsible for these phenomenons remain to be not yet clear. To better serve the discovery of potential strategies for the treatment of oral cancer, we reviewed the effects of MSCs on oral cancer, the underlying mechanisms, and MSCs’ potential applications in the treatment of oral cancer.

## 2 Discovery and Characterization of Mesenchymal Stem Cells

Originated from the mesoderm and firstly found in bone marrow, MSCs can be isolated from multifarious post-natal tissues, including adipose, umbilical cord, umbilical cord blood, amniotic fluid, and other tissues (Whiteside, 2018; Almeida-Porada et al., 2020; Tavakoli et al., 2020). Apart from these tissues, MSCs have also been isolated from dental tissues, including dental pulp, dental follicle, apical papillae, periodontal ligament, and gingiva (Shi and Gronthos, 2003; Pierdomenico et al., 2005; Janjanin et al., 2008; Lindroos et al., 2008; Sonoyama et al., 2008). MSCs are positive for CD105, CD90, CD73, CD146, CD29, STRO-1, but are negative for CD14, CD34, CD45 (Lei et al., 2014; Hung et al., 2015; Ji et al., 2016). Under suitable inductive medium, MSCs are capable of differentiating into osteoblasts, adipocyte, chondrocytes, and many other cells (Whiteside, 2018).

MSCs are localized throughout the adult body as a small population in the stroma of the tissue concerned, and the micro-environment protect their self-renewal potential and undifferentiated state (Urbanek et al., 2006; Wang et al., 2011). Upon tissue injury or inflammation insult, MSCs are activated and leave their ecological niche and migrate to the site of injury, inflammation and tumors, where they are able to secrete various cytokines, chemokines, and growth factors that closely interact with the inflammatory environment and the tumor environment (TME), respectively (Sun et al., 2014).

Compared to embryonic stem cells, MSCs are related with fewer ethical issues, and have emerged as one of the most promising cell therapy tools due to their excellent biological properties, such as relatively simple cell isolation procedure, high potential for expansion, low immunogenicity, pluripotency, and the ability to secrete mediators that support tissue transformation or replacement (McLeod and Baylis, 2006; Caulfield and Ogbogu, 2011; Widdows et al., 2014; Kariminekoo et al., 2016; Musiał-Wysocka et al., 2019). With the accumulation of data about the interaction between MSCs and tumor cells, MSCs have been demonstrated the natural anti-tumor functions, which is the basis for intensive research for new methods using MSCs as a tool to inhibit tumor growth and invasion, although the dualistic role of MSCs on tumors still exists.

## 3 Interaction Between Mesenchymal Stem Cells and Tumors

**FIGURE 1:**
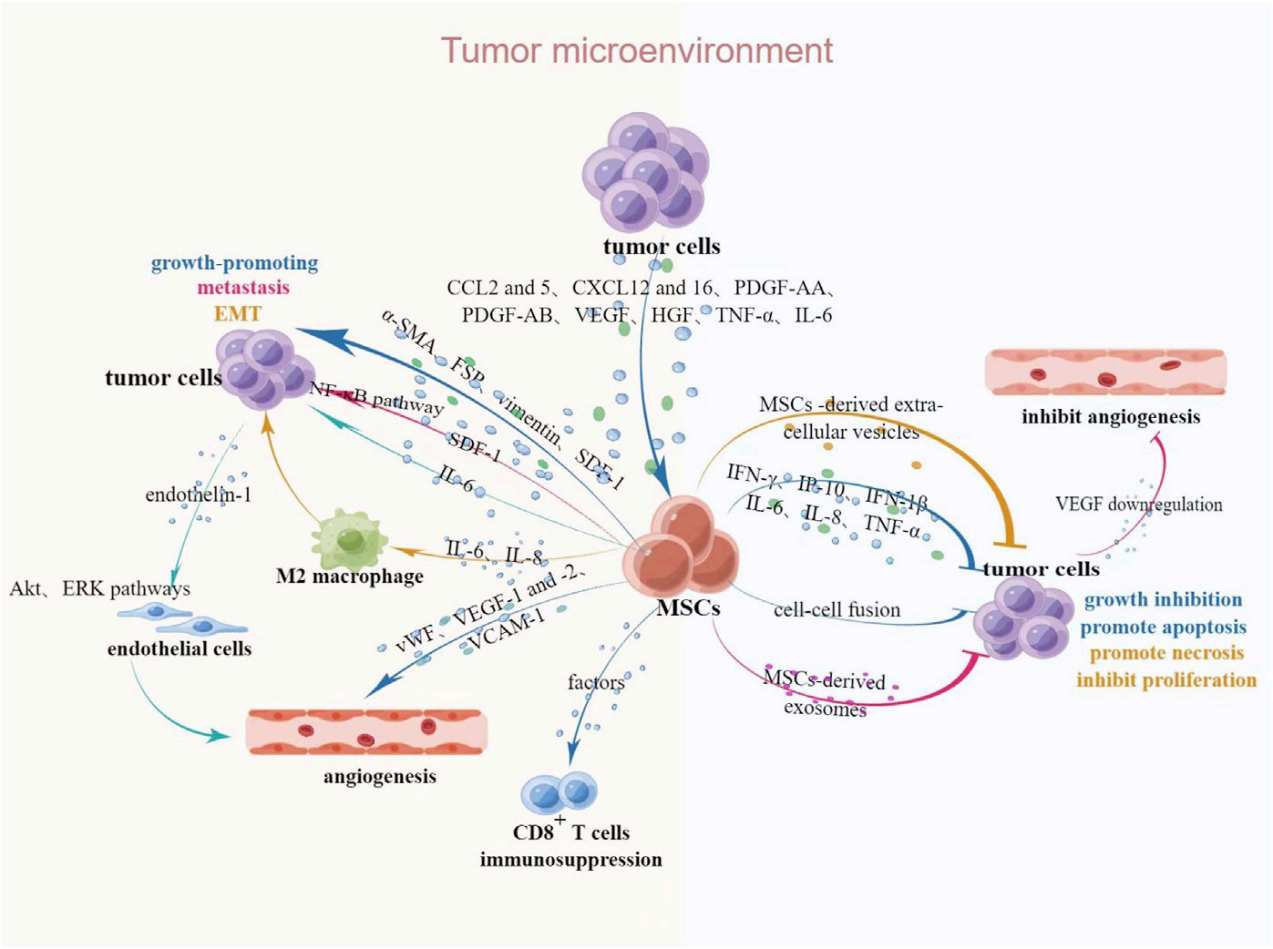
Interaction between MSCs and tumors in TME. Upon tumorigenesis, tumors secrete relevant chemokines, cytokines and growth factors to attract MSCs, after which MSCs undergo a series of phenotypic and functional changes and secrete relevant bioactive factors to act on neighboring cells, thus exerting tumor-promoting or suppressive effects. Abbreviations: CCL2 and 5, CC-chemokine ligands 2 and 5; CXCL12 and 16, chemokine ligands 12 and 16; HGF, hepatocyte growth factor; α-SMA, α-smooth muscle actin; FSP, fibroblast surface protein; SDF-1, stromal-derived factor 1; IP-10, IFN-γ-inducible protein 10. This figure was created by using Figdraw.

The interaction of MSCs and malignant tumors provides new clinical ideas for the treatment of malignant tumors. However, the promotive or inhibitory effects of MSCs on tumors are still inconclusive. It is shown that the normal tissue microenvironment, whose stability is usually maintained by MSCs, is subsequently changed to TME once a tumor develops and the *in vivo* homeostasis is disrupted, and TME also could be remodeled by MSCs of normal tissue origin (Whiteside, 2018). Interactions between MSCs and tumors in TME are described in Figure 1.

Some chemokines, cytokines and growth factors in TME have strong chemotactic effects on MSCs, such as CC-chemokine ligands 2 and 5, chemokine ligands 12 and 16, platelet-derived growth factor (PDGF)-AB, vascular endothelial growth factor (VEGF), hepatocyte growth factor and tumor necrosis factor (TNF)-α (Ponte et al., 2007; Shi et al., 2017). It is reported that after exposure to tumor cell-conditioned medium (CM), bone marrow derived MSCs (BMSCs) acquired a myofibroblast phenotype, which characterized by increased expression of α-smooth muscle actin, fibroblast surface protein, vimentin and stromal-derived factor 1, thereby promoting the growth of tumor cells (Mishra et al., 2008). After co-culture with VEGF, MSCs acquired a lymphatic phenotype and an endothelial cells phenotype characterized by the expression of von Willebrand factor, VEGF receptors 1 and 2, vascular endothelial-keratin, and vascular cell adhesion molecule-1, which promote vascular and lymphatic angiogenesis, ultimately lead to regional lymph node metastasis (Oswald et al., 2004; Conrad et al., 2009). These effects are also verified by the study that the BMSCs-mediated tumor promotion is associated with enhanced angiogenesis induced by the secretion of pro-angiogenic factors. In a study of colorectal cancer, Huang et al. (2013) found that MSCs-derived IL-6 could activate Akt and ERK pathways in endothelial cells through upregulation of endothelin-1 in cancer cells, which subsequently enhanced tropism and angiogenesis to tumors. Stromal-derived factor-1α secreted by BMSCs promotes the invasion of squamous cell carcinoma by activating nuclear factor-κB (Chung et al., 2006; Rehman and Wang, 2008; Xu et al., 2019). The epithelial-mesenchymal transformation (EMT) is a key factor in the migration and invasion of malignant tumors. The morphology and phenotype of cancer cells post-EMT are altered and acquire the ability to metastasize to distant sites. Tumor-derived MSCs secrete large amounts of IL-6 and IL-8 to stimulate M2 macrophage polarization, which enhances the EMT of gastric cancer, thus promoting migration and invasion of tumor (Dominici et al., 2006; Li et al., 2019b). MSCs have been reported to favor tumor growth due to immunosuppression, and Djouad et al. (2003) demonstrated that soluble factors produced by MSCs exert immunosuppressive effects by mediating the inhibition of lymphocyte proliferation by CD8^+^ regulatory cells, and further identified the tumor growth-promoting effect of MSCs in a study of B16 melanoma.

In contrast with the above results, a growing number of studies have identified a dual role of MSCs for tumor progression. Pakravan et al. (2017) demonstrated that exosomes derived from BMSCs were able to downregulate the expression of VEGF in breast cancer cells, thereby inhibiting vascular growth. It is reported that interferon (IFN)-γ, IFN-γ-inducible protein 10, IFN-1β, IL-6, IL-8, and TNF-α appear to play an important role in the inhibitory effect of MSCs on tumor cells (Tomchuck et al., 2008). MSCs may inhibit tumor progression by means of cell fusion. Wang et al. (2012b) successfully inhibited the progression of esophageal cancer by inhibiting tumor cells growth and increasing apoptosis by fusing human umbilical cord MSCs with esophageal cancer cells. Human BMSCs derived extracellular vesicles can promote apoptosis or necrosis and inhibit the proliferation of tumor cells in hepatocellular carcinoma, ovarian cancer and Kaposi’s sarcoma by activating cell cycle negative regulators (Bruno et al., 2013).

Given the complex interactions between MSCs, tumor cells, and TME, no simple factor by itself can determine the fate of tumor, and further studies are needed to better understand the special functions of MSCs in the TME before these cells become valuable tools in cancer therapy.

## 4 Effects of Mesenchymal Stem Cells on Oral Cancer

In recent years, there have been gradually increasing studies on the relationship between the MSCs and oral cancer, and in these studies, it was found that, on the one hand, MSCs have an inhibitory effect on oral cancer, while on the other hand, MSCs can promote the progression of oral cancer. In addition, the effects of MSCs derived from different sources on oral cancer were not consistent. The effects of different MSCs on oral cancer are shown in Table 1.

**TABLE 1:** Effects of MSCs on oral cancers.

MSCs sources	Types of cell lines	*In vitro*	Culture patterns	*In vitro* effects	*In vivo*	Animal models	*In vivo* effects	Underlying mechanisms	References
Human deciduous exfoliated teeth	CAL27	—	—	—	√	BALB/C nude mice, multi-point intratumoral injection	Decreased the volume of tumor and the production of microvessels around the tumor	Downregulation of VEGF-A expression through miR-100-5p and miR-1246	Liu et al. (2022)
Human dental pulp	AW13516	√	CM from MSCs + tumor cells	Augmented the carcinogenic properties of tumor cells	—	—	—	Alterment of growth factors, pro-inflammatory cytokines, and anti-inflammatory cytokines	Raj et al. (2021a)
Human dental pulp	AW13516	√	CM from MSCs + tumor cells	Promoted proliferation of tumor cells at low concentrations and inhibited proliferation at high concentrations	—	—	—	Alterment of pro-inflammatory cytokines (TNF-mmatory cytokinesmmatory cytokinescentrations and inhibited proliferation at PDGF-BB)	Raj et al. (2021b)
Human gingival tissue	CAL27,WSU-HN6	√	Cell-HN6ngival ti co-culture, CM from MSCs + tumor cells, transwell co-culture	Inhibited the growth of tumor cells	√	BALB/c nude mice injected subcutaneously	Inhibited the growth of tumor	Activation of the JNK signaling pathway	Ji et al. (2016)
Human bone marrow	HSC-3	√	Cell–cell contact co-culture, CM from MSCs + tumor cells，3D organotypic model	Inhibited the proliferation but increased the invasion of tumor cells	—	—	—	Upregulation of CCL5 and type I collagen mRNA	Salo et al. (2013)
Human bone marrow	CAL27	√	CM from MSCs + tumor cells	Promoted the proliferation, migration, EMT, and alterd the expression of cell cycle regulatory proteins and inhibition of apoptosis of tumor cells	√	BALB/C nude mice, injected into the middle of the tongue	Promoted the growth, invasion, metastasis of tumor and enhanced the expression of POSTN and EMT in tumor	Activation of POSTN-mediated PI3K/Akt/mTOR signaling pathway	Liu et al. (2018)
Human bone marrow	CAL27	√	MSCs + CM from tumor cells，CM from MSCs+tumor cells，transwell co-culture	Recruited by tumer cells，promoted the proliferation，migration and inhibition the apoptosis of tumor cells	√	BALB/c nude mice, injected intravenously via tail vein	Promoted tumorigenesis and EMT	CXCL8-CXCR2，TGF-β1/Ras/Raf/Erk	Meng et al. (2020)
Mice bone marrow	OSCC cell line (moderately differentiated tumor of buccal mucosa)	—	—	—	√	NU/NU nude mice, injected directly intra-tumorally, combination therapy with cisplatin	Reduced inflammation at the tumor site, increased microvascular production, minimized hypoxia , and reduced tumor growth by promoting tumor cell apoptosis	—	Zurmukhtashvili et al. (2020)
Human bone marrow	TCA8113	√	Exosomes derived from MSCs + tumor cells, transwell co-culture	Exosomes transfer miR-101-3p to tumor cells and then inhibited the proliferation, invasion and migration of tumor cells	√	BALB/c nude mice, injected subcutaneously	Inhibited the growth of tumor	Downregulation of miR-101-3p to COL10A1	Xie et al. (2019)
Human bone marrow	CAL27	√	CM from MSCs + tumor cells	Induced apoptosis and reduced the viability and proliferation of tumor cells	—	—	—	—	Bagheri et al. (2021)
Human amniotic membrane	CAL27	√	CM from MSCs + tumor cells	Inhibited apoptosis and promoted the viability and proliferation of tumor cells	—	—	—	—	Bagheri et al. (2021)
Rat tumor	Oral mucosa malignancy induced by 4-nitroquinoline-1-oxide	√	Anti-CD3 pre-stimulated rat splenocytes + tumor derived-MSCs	Inhibited T cells proliferation but did not affect apoptosis nor migration	—	—	—	Inhibition of T cells proliferation	Chen et al. (2019)
Human oral leukoplakia with dysplasia and OSCC	SCC15	√	Cell–cell contact co-culture,3D coculture model	Promotes the proliferation, migration and invasion of tumor cells	—	—	—	Upregulation of microRNA-8485	Li et al. (2019a)
Human TSCC	CAL27, WSU-HN6	√	CM from MSCs and tumor cells, transwell co-culture	Promoted the metastasis of tumor cells by EMT	√	BALB/c nude mice, injected intravenously via tail vein	Promoted the metastasis of tumor cells	Activation of the NF-κB signaling pathway	Ji et al. (2021)
Human adipose tissue	HSC-3	√	CM from MSCs + tumor cells, MSCs + CM from tumor cells	Did not enhance the invasive or migratory potential of tumor cells	—	—	—	—	Sinha et al. (2020)

Different MSCs could promote or inhibite oral cancers, which were summed up in Table 1, including MSCs sources, types of cell lines, *in vitro* culture patterns, *in vitro* effects, *in vivo* animal models, *in vivo* effects, underlying mechanisms, and references.

### 4.1 Dental Tissues Derived Mesenchymal Stem Cells (DMSCs)

Ji et al. (2016) showed that gingival tissue derived MSCs (GMSCs) inhibited the growth of tongue squamous cell carcinoma (TSCC) cell lines CAL27 and WSU-HN6 *in vitro*, and the soluble factors in CM of GMSCs played a key role in this process by inducing apoptosis of tumor cells. GMSCs could upregulate the expression of the apoptotic associated genes Bax, cleaved poly ADP-ribose polymerase, cleaved caspase-3, and downregulate the proliferation related genes, including Bcl-2, phosphorylation extracellular signal-regulated kinase 1/2, cyclin dependent kinase 4, cyclin D1, proliferating cell nuclear antigen and survivin, which indicating that GMSCs could inhibit the growth of tongue TSCC by activating the JNK signaling pathway. In addition, subcutaneous injection of GMSCs in nude mice model significantly inhibited the growth of tumors.

As mentioned earlier, MSCs can influence tumor progression by regulating intra-tumor angiogenesis. Anti-angiogenic therapy may be a possible treatment strategy of tumors in the future. Liu et al. (2022) treated TSCC CAL27 cells with exosomes derived from human deciduous exfoliated teeth by direct multi-point intratumor injection and found that the tumor volume was significantly smaller than that of the control group. Further experiments showed that exosomes from human deciduous exfoliated teeth downregulated VEGF-A expression through miR-100-5p and miR-1246, which significantly reduced the generation of microvasculature around TSCC.

Dental pulp stem cells (DPSCs)-derived CM induced apoptosis in tumor cells, inhibited the proliferation of TSCC cells AW13516 by enhancing the expression of p16, enhanced the invasion, adhesion, and multidrug resistance of AW13516 by upregulating angiopoietin-2, epidermal growth factor, macrophage-stimulating factor, PDGF-AA, PDGF-BB, TNF-α, and IL-2, downregulating the anti-inflammatory cytokines TNF-β1, and pro-inflammatory cytokine IL-4 (Raj et al., 2021a).

Based on Ki-67 assays, Raj et al. (2021b) indicated the dual effect of MSCs on tumors. DPSCs-CM inhibited the proliferation of TSCC cells AW13516 at 50% and 100% concentrations and promoted the proliferation at a 20% concentration. Several growth factors, including VEGF, hepatocyte growth factor, angiopoietin-2, transforming growth factor (TGF)-α, stem cell factor, erythropoietin, colony-stimulating factor, fibroblast growth factor, and PDGF-BB, and pro-inflammatory cytokines, TNF-α and IL-8, may play a dominant role in promoting the proliferation of tumor cells.

### 4.2 BMSCs

#### 4.2.1 Promotional Effects of BMSCs on Oral Cancer

Human BMSCs inhibited the proliferation of TSCC cells HSC-3, but promoted the invasion of tumor cells by upregulating the expression of the C-C Motif Chemokine 5. In addition, BMSCs-induced production of type I collagen after interaction with HSC-3 is associated with poor prognosis in TSCC cells patients (Salo et al., 2013).

Human BMSCs-CM was demonstrated to promote the expression of periostin, and further contribute to CAL27 progression through the activation of the phosphoinositide 3-kinase /Akt/mammalian target of rapamycin signaling pathway. In a murine model of TSCC, the authors found that BMSCs promoted tumor growth, invasion, metastasis and enhanced the expression of periostin in tumor tissues. After co-cultured with BMSCs-CM, CAL27 cells’ expressions of Snail, Twist, N-cadherin and vimentin were significantly increased, and the expression of E-cadherin was significantly decreased, which suggests that BMSCs could promote EMT of TSCC cells (Liu et al., 2018).

A series of *in vitro* and *in vivo* experiments showed that C-X-C chemokine receptor 2 expressed by BMSCs combined with IL-8 expressed by CAL27 promoted the migration of BMSCs to the tumor stroma. The TGF-β/rat sarcoma virus/rapidly accelerated fibrosarcoma/extracellular signal-regulated kinase pathway activated by BMSCs promoted EMT of TSCC, thereby promoting its proliferation, migration and infiltration (Meng et al., 2020).

#### 4.2.2 Inhibitory Effects of BMSCs on Oral Cancer

By using hamsters OSCC model, Bruna et al. (2018) demonstrated that systematic administration of allogeneic BMSCs did not aggravate the progression of precancerous lesions. On the contrary, administration of BMSCs at the hypoplasia stage of precancerous lesions inhibited tumor growth, whereas only a small proportion of lesions transformed to OSCC by applying BMSCs at the papilloma stage.

Xie et al. (2019) demonstrated that human BMSCs could transfer microRNA-101-3p to human TSCC cells TCA8113 *via* exosomes, and collagen type X alpha 1 chain is negatively regulated by microRNA-101-3p as its target gene. By down-regulating collagen type X alpha 1 chain, microRNA-101-3p could inhibit the proliferation, invasion and migration of TCA8113 cells. Subcutaneous injection of human BMSCs-microRNA-101-3p in nude mice revealed a significant reduction in tumor volume and weight, confirming the inhibitory effect of BMSCs derived microRNA-101-3p on tumor growth *in vivo*.

Bagheri et al. (2021) demonstrated that human BMSCs-CM exhibited a time-dependent inhibitory effect on TSCC cells CAL27, which showed the lowest cells viability after 72 h of co-culture with BMSCs-CM. The decrease of proliferation marker proliferating cell nuclear antigen, anti-apoptotic marker BCL-2 and Ki67 positive cells at 24 and 72 h of co-culture indicated that BMSCs-CM decreased the proliferation of CAL27 cells and increased apoptosis.

Another study demonstrated that 3 weeks after oral tissue injection of human OSCC (moderately differentiated tumor of buccal mucosa) in nude mice, direct intra-tumor injection of mice-derived BMSCs in combination with cisplatin revealed an inhibition of tumor growth and an increase in the lifespan of the mice, these effects may be due to BMSCs’ promotion of cisplatin distribution for better anti-cancer action and increased apoptosis of tumor cells (Zurmukhtashvili et al., 2020).

### 4.3 Other Mesenchymal Stem Cells

Among the applications of MSCs for cancer treatment, adipose tissue derived MSCs (AMSCs) have received increasing attention due to the advantages of relatively easy collection and production. Sinha et al. (2020) demonstrated that after co-culture with human TSCC cell HSC-3, AMSCs did not induce proliferation, migration, and invasion of tumor cells, which providing preliminary evidence that AMSCs may have more suitable properties for tumor therapy relative to other types of MSCs. Because there are relatively few studies on interaction of AMSCs and tumors, more studies are needed before further applications.

In a study of human amniotic membrane MSCs (HAMCSs)-derived CM, its promoting effect on TSCC cells CAL27 was demonstrated after a 24 h of co-culture with human HAMSCs-CM, manifested by an increase in CAL27 cell viability. After 72 h of co-culture, the increased expression of proliferating cell nuclear antigen indicated that HAMSCs-CM promoted the proliferation of CAL27, and the results of flow cytometry showed a decrease in the number of apoptotic CAL27 cells (Bagheri et al., 2021).

Chen et al. (2019) demonstrated that rat oral mucosa malignancy-derived MSCs can inhibit the proliferation of T cells and promote the apoptosis of T cell through soluble factors and intercellular contacts, whereas T cell migration was not affected. The immunosuppressive effect of MSCs on T cells is enhanced with increasing tumor malignancy. The higher the number of MSCs at the tumor sites, the higher the proliferative status of tumor cells, showing that tumor-derived MSCs play an important role in the malignant progression of oral mucosa.

Increased expression of microRNA-8485 in exosomes derived from human oral leukoplakia with dysplasia and oral cancer (species not specified) derived-MSCs promotes the proliferation, migration and invasion of SCC15 cells *in vitro* and reduces the expression of the oncogene p53 (Li et al., 2019a). Ji et al. (2021) showed that OSCC-derived MSCs promoted the migration and invasion of OSCC cell lines CAL27 and WSU-HN6. With a significant decrease in E-cadherin, alpha E catenin and a significant increase in N-cadherin, OSCC-MSCs may promote OSCC metastasis through EMT. Further experiments showed that upregulation of CPNE7, a calcium-dependent phospholipid -binding protein in tumor-derived MSCs, promotes phosphorylation of p65 and IκBα as well as nuclear translocation of p65, which activates the NF-κB pathway, promotes the expression of IL-8, and thus promotes tumor metastasis.

## 5 Treatment of Oral Cancer

### 5.1 Current Status of Oral Cancer Treatment

The treatment of oral cancer depends mainly on the severity of the disease. OSCC is the most common type of oral cancers with a poor prognosis and a high recurrence rate (Satija et al., 2009) and a huge potential for regional metastasis even in the early stages (Vargas-Ferreira et al., 2012). A proportion of OSCC can be detected at an early stage, but current treatment modalities adversely affect patients physically and psychologically, severely affecting their quality of life. Most OSCC is not detected until advanced stage, by which time the survival rate of patients has been markedly reduced. Among the many treatment modalities, surgical treatment is the main modality for oral cancer. When the primary tumor is large and incomplete resection or signs of infiltration are suspected, radiation adjuvant therapy is administered after surgery. Molecularly targeted therapy with cetuximab added to postoperative radiotherapy, targeting the epidermal growth factor receptor, has been approved for the treatment of OSCC. Docetaxel, cisplatin and 5-fluorouracil and other anticancer drug-induced chemotherapy are usually used as induction therapy before surgery or alone or in combination with radiotherapy after surgery. Combined surgery-radiotherapy has become the standard procedure for the treatment of advanced oral cancer (Colevas et al., 2018). In recent years, antitumor immunotherapies such as the programmed death-1 inhibitor, which block tumor immunosuppressive signals and enhance antitumor immune responses by targeting the programmed death-1/ programmed death-ligand 1 pathway, have played an important role in recurrence and metastasis oral cancer (Cramer et al., 2019).

Although currently available treatment strategies include excision of malignant tissue in combination with radiotherapy and chemotherapy, the 5-years survival rate is still about 50% (Sasahira et al., 2014). The ultimate goal of surgery is to remove the tumor tissue, but inadequate removal has a high chance of causing recurrence (Brennan et al., 1995). The oral cavity as a functional organ of mastication, speech, and articulation, surgery may lead to serious aesthetic and functional problems, as well as psychological trauma for the patient. Radiotherapy can cause altered taste, dysphagia, dry mouth, and hypothyroidism, causing temporary or permanent damage to healthy tissues. Chemotherapy can also lead to severe systemic reactions such as nausea, vomiting, hair loss, infection and diarrhea, which can seriously affect patients’ health and quality of life. Therefore, it is particularly important to find new methods for the treatment of oral cancer.

As previously mentioned, MSCs can inhibit tumor progression in multiple ways, such as inhibition of angiogenesis, suppression of cell proliferation and metastasis, induction of apoptosis, cell cycle arrest, inflammatory infiltration, and regulation of oncogenes. In light of these studies on MSCs, there has been increasing interest in MSCs-based cancer therapy in recent years, and advanced approaches to modify MSCs to become powerful and precise targeting tools for killing cancer cells rather than normal healthy cells have been continuously explored.

### 5.2 Mesenchymal Stem Cells in the Treatment of Oral Cancer

As important participants in TME remodeling, the interaction between MSCs and tumors begins at the early stage of tumor growth, MSCs are attracted to the tumor sites and interact with surrounding cells, undergoing phenotypic and functional changes and secreting a variety of bioactive factors that can significantly alter the growth, proliferation, and apoptosis of neighboring cells, thereby affecting tumor progression. As mentioned earlier, MSCs show both promoting and inhibiting effects on oral cancer, therefore, their mechanisms should be further explored before applying MSCs to the treatment of oral cancer. In addition, circumventing the promotive effects of MSCs on oral cancer and making full use of the effects of MSCs on oral cancer, such as homing and inhibition, and using them as therapeutic drug carriers or MSCs to directly inhibit tumors would be beneficial to discover the potential applications of MSCs for oral cancer treatment.

Both wild-type MSCs as well as modified MSCs have been used for the treatment of oral cancer, as shown in Table 2. MSCs can be used as carriers for delivery of therapeutic proteins or anticancer drugs, and are genetically modified to over-express several anti-tumor factors, such as IL, IFN, pro-drugs, oncolytic viruses, pro-apoptotic proteins, anti-angiogenic agents and growth factor antagonists (Shah, 2012). MSCs can selectively migrate and aggregate at the tumor sites, thus exerting a therapeutic effect, improving therapeutic efficacy and reducing systemic toxicity.

**TABLE 2:** MSCs-mediated treatment of oral cancers.

MSCs sources	Types of cancer/cell lines	Type of studies	Application methods for MSCs	Outcome effects	References
Human deciduous exfoliated teeth	CAL27	*In vivo*	Multipoint intratumoral injection	Decreased the volume of tumor and the production of microvessels around the tumor	Liu et al. (2022)
Human bone marrow	TCA8113	*In vitro* and *in vivo*	Injected subcutaneously	Exosomes transfer miR-101-3p to tumor cells and then inhibited the proliferation, invasion and migration of tumor cells	Xie et al. (2019)
Hamster bone marrow	OSCC induced by mineral oil or DMBA	*In vivo*	Injected around the tumors	MSC administration at papilloma stage precludes tumor growth and epithelial dedifferentiation of OSCC	Bruna et al. (2017)
Mice bone marrow	OSCC (moderately differentiated tumor of buccal mucosa)	*In vitro*	Injected directly intratumorally	Reduced inflammation, increased micro-vascularization, and minimize hypoxia of orthotopic tumor tissues. Combined treatment with Cisplatin leaded to higher apoptotic activity and reduced tumor tissue growth	Zurmukhtashvili et al. (2020)
MSCs (species not specified)	OSCC	*In vivo*	Sonodynamic Treatment, M/LPV/O2	Induced tumor cells death, displayed good tumor accumulation and penetration under ultrasound stimulation, and efficiently induces tumor inhibition and even abrogation, exhibited minimal systemic adverse effects and successfully maintained oral functions with no facial tissue damage	Sun et al. (2020)
Mice bone marrow	Oral potentially malignant disorders	*In vivo*	BMSCs-EVs-miR-185, directly pasted	Reduced inflammatory conditions and dysplasia of diseased tissue, inhibited proliferation, angiogenesis and promoted activation of the Akt pathway to increase apoptosis	Wang et al. (2019)
Human gingival papilla	SCC154	*In vitro*	Cell-mediated drug delivery system	GinPa-MSCs effectively binded the drug and released it in an active form and in sufficient quantity to significantly inhibited the growth of tumor	Coccè et al. (2017)
Human gingival tissue	TSCC (TCA8113 and CAL27)	*In vitro*, *In vivo*	Vehicle for cell-based gene therapy, GMSCs with full-length TRAIL, mixed injection with tumor cells and tail vein injection	Induced massive necrosis and apoptosis of tumor cells *in vitro*, GMSCFLT reduced or even inhibited the growth of TSCC *in vivo*	Xia et al. (2015)
Human gingival tissue	TSCC (CAL27)	*In vitro*, *In vivo*	GMSCs genetically engineered to produce IFN-β as a targeted gene delivery system (GMSCs/IFN-t), injected subcutaneously	Inhibited the proliferation of CAL27 cells *in vitro*, inhibited the growth of tumor by suppressing cell proliferation and inducing apoptosis *in vivo*	Du et al. (2019)
Human dental Pulp	TSCC (CAL27)	*In vitro*, *In vivo*	Modification of Metal-Organic Framework Nanoparticles Using MSCs Membranes to Target tumor (MOF@DPSCM)	Inhibited the growth of OSCC *in vitro* and *in vivo*	Zhou et al. (2021)

The therapeutic effects of MSCs on oral cancers were summed up in Table 2, including MSCs sources, types of cancer/cell lines, types of studies, application methods for MSCs, therapeutic effects, and references.

Unlike cell therapies, MSCs derived secretomes can be better evaluated for their safety, dosage and potency. Secretomes, aside from avoiding the inconveniences of administering living proliferating cells, show other additional advantages, including cheaper, safer, and more practical for clinical use. For instance, exosomes derived from MSCs, one of the secretomes, have been evaluated for their potential to be used as drug delivery vehicles (Eiro et al., 2021). The treatments of different MSCs on oral cancer are shown in Table 2.

#### 5.2.1 Direct Application of Mesenchymal Stem Cells

Isolated from gingival tissues, GMSCs possessed the properties of easy isolation, rapid expansion, profound immunomodulatory and anti-inflammatory functions, making them potential source for stem cell-based therapy (Ji et al., 2016). GMSCs can inhibit the growth of oral cancer cells *in vitro* and *in vivo* by altering the microenvironment of surrounding oral cancer cells, suggesting that GMSCs have potential applications in the treatment of oral dysplasia and oral cancer (Ji et al., 2016).

MSCs can influence tumor progression by regulating intra-tumor angiogenesis. Anti-angiogenic therapy may be a possible treatment strategy of tumors in the future. Liu et al. (2022) treated TSCC CAL27 cells with exosomes derived from human deciduous exfoliated teeth by direct multi-point intratumor injection and found that the tumor volume was significantly smaller than that of the control group. Further experiments showed that exosomes from human deciduous exfoliated teeth downregulated VEGF-A expression through miR-100-5p and miR-1246, which significantly reduced the generation of microvasculature around TSCC.

Up to date, the influence of BMSCs on cancer remains uncertain. Some studies have shown that BMSCs promote cancer progression, whereas others show that BMSCs suppress cancer progression, and also some studies found BMSCs have no significant impact on cancer progression (Barcellos-de-Souza et al., 2013; Gao et al., 2016; Mi and Gong, 2017; Wu et al., 2019; Zhang et al., 2019). By establishing an animal model, Bruna et al. (2017) showed that the administration of BMSCs at the precancerous stage (papilloma stage) reduced the proliferation rate of OSCC tumors and increased the apoptosis rate, thereby stopping tumor growth and preventing epithelial dedifferentiation of OSCC. Zurmukhtashvili et al. (2020) established an OSCC model in mice and demonstrated that intratumorally injected BMSCs reduce inflammation, increase micro-vascularization, and minimize hypoxia of OSCC tissues. Moreover, combined treatment with cisplatin leads to higher apoptotic activity and reduced tumor tissue growth.

#### 5.2.2 As Drug Delivery Vehicles

GMSCs exhibit the capability to encapsulate and release anticancer drugs without any genetic changes. Coccè et al. (2017) demonstrated by *in vitro* experiments that GMSCs can efficiently bind three important antitumor drugs and then release them in active form: paclitaxel, doxorubicin, and gemcitabine, thereby significantly inhibiting the growth of TSCC cell SCC154, indicating that MSCs-mediated drug delivery systems have potential applications in the field of oral oncology.

Zhou et al. (2021) showed that OSCC recruits DPSCs through the CXCL8-CXCR2 axis. They used DPSCs membranes (DPSCM) modified with metal-organic framework nanoparticles (MOFs) to create a novel nanoparticle, MOF@DPSCM, which can effectively deliver antitumor drugs to target OSCC. MOF@DPSCM carries doxorubicin (DOX) can induce the death of CAL27 cells and block the growth of CAL27 tumor. The data suggests that this novel MOF-DOX@DPSCM nanoparticle is a potential targeted drug delivery system that can be used for OSCC.

Sun et al. (2020) used MSCs membrane-encapsulated oxygen-carrying perfluorocarbon and sonosensitizer verteporfin to develop a novel biomimetic sonosensitizer, named M/LPV/O_2_, which could induce cancer cell death by increasing uptake cancer cells and stimulating intracellular reactive oxygen species production under hypoxic conditions. M/LPV/O_2_ can accumulate in oral tumor, relieved hypoxia, and effectively inhibited tumor. In addition, exhibiting minimal systemic side effects, M/LPV/O_2_ can successfully maintain oral function without causing aesthetic problems.

#### 5.2.3 Expression of Anti-Tumor Factors by Gene Modification

Tumor necrosis factor-related apoptosis-inducing ligand (TRAIL) is a promising target that can selectively induce apoptosis in cancer cells. Demonstrating *in vitro* that human GMSCs can migrate to TSCC cell lines (TCA8113 and CAL27), Xia et al. (2015) used GMSCs as a cellular vector and transduced with full-length TRAIL (GMSCs with full-length TRAIL; GMSCFLT), and found that GMSCFLT induced massive necrosis and apoptosis of tumor cells by co-culture with TCA8113 and CAL27, respectively. In addition, further antitumor assays using GMSCFLT tail vein injection into nude mice significantly inhibited the growth of TSCC. These data confirmed the tumor suppressive effect of gene therapy vectors TRAIL-expressing GMSCs.

Previous studies demonstrated the inhibitory effect of IFN-β gene-modified MSCs on breast, pancreatic and prostate cancers (Kidd et al., 2010; Ling et al., 2010; Wang et al., 2012a). Du et al. (2019) constructed IFN-β gene-modified GMSCs (GMSCs/IFN-β), and found that GMSCs/IFN-β inhibited the proliferation of CAL27 cells *in vitro*. Through *in vivo* experiments, they showed that GMSCs/IFN-β expressed high levels of IFN-β, which significantly inhibited tumor growth. Further experiments demonstrated that GMSCs/IFN-β inhibited the growth of TSCC xenografts by suppressing cell proliferation and inducing apoptosis. In addition, GMSCs are genetically modified to release other cytokines, such as IFN-γ, IL-2, IL-12, and IL-24 (Matsuzuka et al., 2010; Zhang et al., 2013; Yang et al., 2014; You et al., 2015), to achieve antitumor effects.

As previously mentioned, exosomes from human BMSCs with upregulated miR-101-3p may serve as a promising new direction for the development of oral cancer therapeutics. Human BMSCs can transfer microRNA-101-3p to human TSCC cells TCA8113 *via* exosomes to negatively regulate the collagen X-type α1 chain of the target gene, thereby inhibiting the proliferation, invasion and migration of TCA8113 cells (Xie et al., 2019).

Oral potentially malignant disorders are usually asymptomatic clinical lesions that appear prior to OSCC (Wang et al., 2019). Put mice genetically modified BMSCs-derived extracellular vesicles (EVs) highly expressing microRNA-185 (BMSCs-EVs-miR-185) pasted on oral potentially malignant disorders induced with dimethylbenzanthracene, and found that BMSCs-EVs-miR-185 could attenuate the inflammatory condition and reduce the dysplasia in the lesion tissue. In addition, BMSCs-EVs-miR-185 may inhibit disease progression by suppressing proliferation, angiogenesis and promoting the activation of the Akt pathway to increase apoptosis of tumor cells.

## 6 Concluding Remarks

Currently, MSCs are used in clinics for anti-inflammatory therapy, tissue regeneration, graft-versus-host response, autoimmune diseases and gene transfection. In addition, MSCs-based tumor therapy is promising, as MSCs can migrate directionally to target tissues to exert their own effects, and can also exert the effects of therapeutic factors by means of gene transfection and secretion of several kinds of factors, presenting the anti-tumor effects.

Under normal conditions, MSCs maintain a stable internal environment in tissues, and once a tumor develops, the normal tissue microenvironment is subsequently changed to a TME. Tumor-secreted cytokines and growth factors, such as PDGF-AB, VEGF, hepatocyte growth factor, IL-8, etc., can recruit local and distant MSCs, and MSCs can also influence tumor progression by affecting angiogenesis, regulating immunity, inducing apoptosis, inhibiting or promoting proliferation, invasion, migration, etc. As mentioned above, MSCs from different tissues have different effects on oral tumors, including those from normal, precancerous and cancerous tissues, which provide a new perspective for further exploration of oral cancer progression and treatment. At the same time, administration of different doses of MSCs may also produce different results. In addition, it seems that different results can be obtained by using different cancer models and by different routes of administration of MSCs at different times of cancer progression. Notably, anticancer treatment can also affect the recruitment of MSCs.

However, since most of the currently available evidence is obtained through non-human xenografts, the literature supporting the direct use of MSCs for the treatment of cancer patients is still insufficient and their safety remains a major consideration, and more research must be conducted to provide evidence and improve the therapeutic efficacy of MSCs in cancer treatment. These cells have great potential to revolutionize existing cancer therapies.
